# Factors Associated with Candidemia After Living Donor Liver Transplantation: A Case–Control Study

**DOI:** 10.3390/jcm14238516

**Published:** 2025-12-01

**Authors:** Mefkure Durmus, Sena Guzel Karahan, Sami Akbulut, Zeynep Burcin Yilmaz, Ertugrul Karabulut

**Affiliations:** 1 Department of Clinical Pharmacy, Karadeniz Teknik University Faculty of Pharmacy, 61000 Trabzon, Türkiye; 2 Department of Clinical Farmacy, Inonu University Faculty of Farmacy, 44280 Malatya, Türkiye; 3 Department of Surgery and Liver Transplantation, Inonu University Faculty of Medicine, 44280 Malatya, Türkiye; 4 Department of Infectious Diseases and Clinical Microbiology, Inonu University Faculty of Medicine, 44280 Malatya, Türkiye

**Keywords:** living donor liver transplantation, candidemia, biliary complications, antibacterial agents, antifungal agents, CMV prophylaxis, immunosuppressive agents

## Abstract

**Background**: Liver transplant recipients are highly susceptible to invasive fungal infections, particularly candidemia, due to intensive immunosuppressive therapy and postoperative complications. However, few studies have comprehensively examined postoperative antimicrobial and immunosuppressive factors in this context. **Aim**: This study aimed to identify perioperative and postoperative factors associated with the development of candidemia in living donor liver transplant (LDLT) recipients, with a particular focus on antimicrobial and immunosuppressive regimens during initial hospitalization. **Methods**: A retrospective case–control analysis was conducted involving 36 LDLT recipients who developed candidemia (candidemia group) and 72 matched controls without candidemia (non-candidemia group) between January 2019 and November 2023. Demographic and clinical variables were compared using univariate and multivariate logistic regression analyses to identify independent associations. A post hoc power analysis demonstrated a high statistical power (97.3%) to detect large effect sizes. **Results**: Univariate analysis revealed significant associations with prolonged intubation (*p* < 0.001), bile leaks (*p* < 0.001), relaparotomy (*p* < 0.001), chronic renal disease (*p* = 0.011), hepatocellular carcinoma (*p* = 0.011), and the use of antimicrobials including meropenem (*p* = 0.048), linezolid (*p* = 0.005), tigecycline (*p* = 0.045), third-generation cephalosporins (*p* = 0.003), anidulafungin (*p* < 0.001), fluconazole (*p* = 0.006), mycophenolate (*p* = 0.011), and total parenteral nutrition (TPN) (*p* = 0.049). CMV prophylaxis (*p* < 0.001) and CMV-PCR positivity (*p* = 0.015) were also significantly associated with candidemia. Multivariate logistic regression analysis identified prolonged intubation (OR = 1.07; *p* = 0.019), bile leaks (OR = 10.9; *p* = 0.002), anidulafungin use (OR = 4.70; *p* = 0.032), fluconazole use (OR = 35.8; *p* = 0.005), and absence of CMV prophylaxis (OR = 11.7; *p* = 0.021) as independent factors associated with increased odds of candidemia. **Conclusions**: Prolonged intubation, bile leaks, antifungal exposure, and lack of CMV prophylaxis are independently associated with higher odds of candidemia after LDLT. Targeted prophylaxis, prudent antimicrobial stewardship, and timely biliary intervention may reduce fungal morbidity and mortality in post-transplant patients.

## 1. Introduction

Liver transplantation (LT) has been established as a life-saving therapeutic procedure for patients with end-stage liver diseases, acute liver failure, pediatric metabolic disorders, and hepatic malignancies [1]. Since Starzl et al. performed the first successful deceased-donor liver transplantation (DDLT) in 1963 [2], the procedure has undergone remarkable evolution. Later, the first living-donor liver transplantation (LDLT) was performed by Raia et al. in 1988 [3], providing an essential alternative for regions with limited deceased organ donation. Over the subsequent decades, survival rates following LT have improved markedly due to refinements in surgical techniques, advances in intensive care unit (ICU) management, and the rational use of immunosuppressive agents [4]. However, with the widespread use of potent immunosuppressive regimens, transplant recipients have become increasingly vulnerable to a spectrum of opportunistic infections, including bacterial, viral, and fungal pathogens [5]. Among these, invasive fungal infections remain particularly concerning due to their high morbidity and mortality rates. LT recipients represent a uniquely immunocompromised population, frequently exposed to multiple risk factors such as prolonged hospitalization, invasive procedures, and broad-spectrum antimicrobial therapy, all of which predispose them to fungal colonization and subsequent infection [6,7].

*Candida* colonization within the human gastrointestinal tract can progress to invasive infection, particularly in patients undergoing abdominal surgery such as LT recipients. In this population, invasive fungal infections predominantly manifest as candidiasis (60–80%), aspergillosis (1–8%), and less frequently as infections caused by other molds or *Cryptococcus* species [8,9,10]. Substantially higher morbidity and mortality rates are observed in cases of invasive aspergillosis (65–90%) and candidiasis (30–50%) [11]. Moreover, the increasing emergence of drug-resistant fungal isolates largely results from excessive or inappropriate antifungal use, underscoring the need for judicious therapeutic stewardship.

Multiple preoperative, intraoperative, and postoperative factors have been implicated in the development of invasive fungal infections among LT recipients [12]. These include elevated Model for End-Stage Liver Disease (MELD) scores, repeated laparotomies, biliary complications, renal replacement therapy, total parenteral nutrition (TPN), pulmonary complications, and prolonged mechanical ventilation [5,13,14,15,16]. The postoperative use of broad-spectrum antibiotics and the occurrence of cytomegalovirus (CMV) infection or viremia have likewise been recognized as important risk factors. Furthermore, the indiscriminate or prolonged administration of antifungal agents has been strongly linked to the emergence of resistant *Candida* species, thereby complicating therapeutic management and increasing the burden of infection [5,16,17,18].

Accordingly, this study sought to elucidate perioperative and postoperative variables associated with the onset of candidemia in LDLT recipients during their index hospitalization. Through a detailed assessment of antimicrobial exposure profiles, immunosuppressive regimens, and infection control strategies, the study aims to generate evidence to inform preventive protocols and enhance antifungal stewardship in the post-transplant setting.

## 2. Material and Methods

### 2.1. Study Period and Place

Data from LT recipients who underwent LDLT at the Inonu University Liver Transplantation Institute (Malatya, Türkiye) between January 2019 and November 2023 were retrospectively analyzed. All medical records were retrieved from the institutional Hospital Information Management System to ensure data completeness and reliability.

### 2.2. Definitions of Study Groups

According to the study’s primary objective, patients were classified into two groups. The case group consisted of LT recipients who developed candidemia during their initial postoperative hospitalization following LDLT (candidemia group; n = 36). The control group included LT recipients who did not develop candidemia during the same period (non-candidemia group; n = 72). Additionally, the cohort was divided into survivors (n = 89) and non-survivors (n = 19) to identify mortality-related factors as a secondary objective.

### 2.3. Sample Size and Power Analysis

This study was designed as a retrospective case–control analysis including all LT recipients who met the inclusion criteria, together with matched controls in a 1:2 case-to-control [19]. This matching ratio has been widely recommended in epidemiological research to improve statistical power while preserving feasibility [19]. The matching process was conducted using a simple randomization technique according to clinically relevant parameters such as age and sex to minimize potential confounding factors. A post hoc power analysis was performed using G*Power software package (Version 3.1.9.6, Franz Faul, Universität Kiel, Germany), based on an assumed effect size (d = 0.8), α = 0.05, and group sizes of 36 and 72 for the candidemia and non-candidemia groups, respectively. The calculated statistical power (1 − β) was 97.3%, and the noncentrality parameter (δ) was 3.9, confirming that the study possessed sufficient statistical strength to detect large effect sizes. A similar post hoc analysis for the survivor and non-survivor comparison yielded a statistical power of 88%, supporting the adequacy of the sample size for secondary outcomes

### 2.4. Inclusion and Exclusion Criteria

The exclusion criteria comprised LT recipients younger than 18 years, those diagnosed with candidemia within 48 h of ICU admission [20,21,22], those diagnosed with candidemia outside their first postoperative hospitalization, and those who underwent DDLT. The inclusion criteria consisted of LT recipients aged ≥ 18 years. As previously stated, recipients in the candidemia and non-candidemia groups were matched based on age and sex using a simple randomization technique. All data were carefully collected to minimize potential bias inherent to the retrospective study design. Cases or controls with incomplete demographic or clinical data were excluded from the final analysis to enhance internal validity.

### 2.5. Data Definitions

For this study, LT recipients in the candidemia group were defined as those who exhibited blood culture positivity for *Candida* spp. following the LDLT procedure during their initial hospitalization [23]. In contrast, LT recipients in the non-candidemia group had no positive blood cultures for *Candida* spp. from the time of LDLT until discharge or in-hospital death. The length of hospitalization was determined as the period between the date of LDLT and either discharge or in-hospital mortality for both groups [24]. Dialysis was defined as the need for hemodialysis or continuous renal replacement therapy, either in the acute or chronic setting [25]. In the candidemia group, patients who received total parenteral nutrition (TPN) for at least two consecutive days within the week preceding the diagnosis of candidemia were recorded as having received TPN. In the non-candidemia group, TPN was considered present if administered for at least two consecutive days at any time during hospitalization. Similarly, in the candidemia group, the presence of a central venous catheter was confirmed if it remained in place for at least three days within the week prior to candidemia diagnosis, whereas in the non-candidemia group, this was recorded if the catheter remained for at least three days during hospitalization [24].

Regarding antibiotic exposure, the candidemia group included patients who had received antibiotic therapy for at least 48 h within the 30 days preceding candidemia diagnosis. For the non-candidemia group, antibiotic use was recorded if it was administered for at least 48 h at any point during hospitalization [23,26,27]. Neutropenia was defined as the presence of two consecutive absolute neutrophil count values below 1500/mm^3^ in patients without preexisting neutropenia [28]. Although many studies rely on a single measurement below this threshold, two consecutive low values were preferred in order to minimize laboratory error and ensure diagnostic reliability. Prolonged intubation was defined according to the Society of Thoracic Surgeons criteria as endotracheal intubation lasting more than 24 h [29,30]. The MELD score was calculated perioperatively to predict survival, using serum bilirubin, creatinine, sodium, and international normalized ratio (INR) values [31].

### 2.6. Postoperative Immunosuppressive Protocol

A standardized postoperative immunosuppressive regimen was implemented for all LT recipients. Methylprednisolone was administered intravenously at a dose of 500 mg following completion of the intraoperative arterial anastomosis. Subsequent doses were tapered gradually: 80 mg twice daily on postoperative day 1, 60 mg twice daily on day 2, 40 mg twice daily on day 3, and 20 mg twice daily on day 4. From postoperative days 5 to 8, 20 mg was given orally once daily; from days 9 to 12, 15 mg once daily; from days 13 to 30, 10 mg once daily; and from months 1 to 3, 5 mg once daily. In patients diagnosed with autoimmune liver disease or those who experienced T-cell–mediated rejection episodes, corticosteroid therapy was continued long term without abrupt discontinuation. During the initial three-month postoperative period, mycophenolate mofetil (MMF) was prescribed at 1000 mg twice daily for recipients weighing more than 60 kg and 500 mg twice daily for those weighing less than 60 kg. Tacrolimus therapy was initiated on postoperative day 3 at a starting dose of 0.10–0.15 mg/kg/day, administered in two divided doses, and titrated to achieve a target serum concentration of 5–12 ng/mL. Cyclosporine and everolimus were introduced as alternative or adjunct agents at initial doses of 10–15 mg/kg (twice daily) and 0.25–0.50 mg (twice daily), respectively. Doses were subsequently adjusted to maintain target serum levels of 100–250 ng/mL for cyclosporine and 3–8 ng/mL for everolimus [32,33]. This protocol ensured consistency in immunosuppressive management across all recipients while allowing individualized adjustments based on postoperative clinical and biochemical findings.

### 2.7. Antimicrobial Prophylaxis Protocol

Prophylactic antimicrobial regimens were applied uniformly to all LT recipients in accordance with institutional guidelines. Trimethoprim–sulfamethoxazole (80/400 mg) was administered for six months to prevent *Pneumocystis jirovecii* pneumonia, and therapy was continued without interruption unless adverse reactions were observed [34]. CMV prophylaxis was achieved with valganciclovir at a dose of 900 mg/day during the first three postoperative months [35,36]. Antifungal prophylaxis was selectively administered to recipients at high risk of invasive fungal infections. High-risk criteria included elevated MELD scores, acute allograft failure, re-transplantation, intraoperative blood transfusion, prolonged operative time, CMV viremia, fungal colonization, renal replacement therapy, prolonged intubation, postoperative biliary complications, or choledochojejunostomy [5]. Recipients meeting any of these risk factors were administered antifungal agents according to established institutional protocols and international recommendations [5]. The infectious diseases team regularly reviewed prophylactic regimens to ensure adherence, limit unnecessary antifungal use, and mitigate resistance development.

### 2.8. Study Protocol and Ethics Committee Approval

This study was conducted in accordance with the principles of the Declaration of Helsinki (1975, as revised in 2013), alongside institutional and national ethical standards governing research involving human subjects. All participants provided both verbal and written informed consent prior to undergoing LDLT, which routinely included permission for surgical intervention and inclusion in scientific research. The study followed the STROBE (Strengthening the Reporting of Observational Studies in Epidemiology) guidelines to ensure methodological transparency, minimize bias, and enhance the reliability of findings [37].

### 2.9. Biostatistical Analysis

All statistical analyses were performed using IBM SPSS Statistics for Windows, Version 25.0 (IBM Corporation, Armonk, NY, USA). The normality of continuous variables was assessed using the Kolmogorov–Smirnov test, and non-normality was confirmed. Continuous variables were expressed as medians with 95% confidence intervals and compared between groups using the Mann–Whitney U test. Categorical variables were presented as frequencies and percentages, and group comparisons were performed using the Chi-squared test. Effect size (ES) was used as a measure of clinical relevance, reflecting the magnitude of intergroup differences. For categorical variables with statistically significant associations, Cramér’s V was calculated and interpreted as follows: negligible (0.00–0.10), weak (0.10–0.20), moderate (0.20–0.40), relatively strong (0.40–0.60), strong (0.60–0.80), and very strong (0.80–1.00). For continuous variables, Cohen’s d was computed and categorized as small (d = 0.2), medium (d = 0.5), or large (d ≥ 0.8) [38]. This approach enabled simultaneous evaluation of both statistical and clinical significance. Odds ratios (ORs) were calculated to determine the strength of association between clinical variables and the presence of candidemia. Because of the case–control design, ORs represented the most appropriate measure of effect and were estimated using univariable and multivariable logistic regression analyses [39,40,41]. Variables identified as statistically and clinically significant in univariable analyses were entered into a multivariable binary logistic regression model. Model calibration was verified using the Hosmer–Lemeshow goodness-of-fit test, and *p*-values < 0.05 were considered statistically significant.

## 3. Results

### 3.1. General Assessment of the Study Cohort

During the five-year study period, a total of 1272 liver transplantations (LTs) were performed, comprising 49 DDLTs and 1223 LDLTs. Among these recipients, 53 (4.16%) were diagnosed with candidemia. Seventeen patients were excluded: eight were younger than 18 years, and nine were not in their initial hospital admission at the time of diagnosis. Notably, none of the recipients in either the candidemia or non-candidemia groups had undergone DDLT. Therefore, 108 LDLT recipients were included in the final analysis: 36 in the candidemia group and 72 in the non-candidemia group.

The distribution of *Candida* species isolated from blood cultures was as follows: *C. albicans* (n = 15; 41.7%), *C. glabrata* (n = 8; 22.2%), *C. parapsilosis* (n = 6; 16.7%), *C. kefir* (n = 4; 11.1%), *C. tropicalis* (n = 2; 5.6%), and *C. gulliermondii* (n = 1; 2.8%). The median interval between antimicrobial use and the first positive blood culture for *Candida* spp. was 25 days (95% CI = 23–28), while the median time from hospitalization to positive culture was 36 days (95% CI = 32–42). Thus, antibiotic therapy was initiated a median of 9 days (95% CI = 3–15) after hospitalization.

### 3.2. Comparison of Candidemia and Non-Candidemia Groups

Table 1 summarizes the comparison of demographic and clinical continuous variables between the candidemia and non-candidemia groups. Prolonged intubation duration was significantly longer in the candidemia group (*p* < 0.001; ES = 0.68). However, no statistically significant differences were observed between the groups in terms of age, MELD score, pre-transplant hospital stay, operation time, cold ischemia time, warm ischemia time, intraoperative crystalloids, blood tacrolimus levels, and postoperative hospital stay, which was a median of 4 days shorter in the candidemia group, although this difference did not reach statistical significance. The shorter postoperative hospital stay observed in the candidemia group may be attributed to the relatively higher mortality rate among these patients.

Table 2 presents the comparison of categorical demographic and clinical variables. Statistically significant differences were found between the groups for bile leaks (*p* < 0.001; ES = 0.38), relaparotomy (*p* < 0.001; ES = 0.39), chronic renal disease (*p* = 0.011; ES = 0.28), hepatocellular carcinoma (*p* = 0.011; ES = 0.28), meropenem use (*p* = 0.048; ES = 0.21), linezolid use (*p* = 0.005; ES = 0.30), tigecycline use (*p* = 0.045; ES = 0.21), third-generation cephalosporin use (*p* = 0.003; ES = 0.31), anidulafungin use (*p* < 0.001; ES = 0.37), fluconazole use (*p* = 0.006; ES = 0.28), lack of CMV prophylaxis (*p* < 0.001; ES = 0.38), MMF use (*p* = 0.011; ES = 0.28), TPN use (*p* = 0.049; ES = 0.20), and CMV PCR positivity (*p* = 0.015; ES = 0.25).

Univariate logistic regression identified bile leaks (OR = 5.5; 95% CI = 2.3–13.4), relaparotomy (OR = 5.7; 95% CI = 2.4–13.7), chronic renal disease (OR = 11.1; 95% CI = 1.2–98.4), hepatocellular carcinoma (OR = 11.1; 95% CI = 1.2–98.4), meropenem use (OR = 2.5; 95% CI = 1.1–5.9), linezolid use (OR = 4.5; 95% CI = 1.66–12.30), tigecycline use (OR = 2.8; 95% CI = 1.1–7.1), third-generation cephalosporin use (OR = 13.7), anidulafungin use (OR = 5.2; 95% CI = 2.1–12.4), fluconazole use (OR = 8.5; 95% CI = 1.7–43.1), absence of CMV prophylaxis (OR = 13.5; 95% CI = 2.8–66.7), MMF use (OR = 11.1; 95% CI = 1.2–98.4), TPN use (OR = 2.4; 95% CI = 1.0–5.6), and CMV PCR positivity (OR = 5.6; 95% CI = 1.3–22.9) as factors significantly associated with increased odds of developing candidemia. Among these, MMF use and CMV prophylaxis appeared to exert protective effects. All other variables listed in Table 2 showed no significant intergroup differences.

Table 3 displays the multivariable logistic regression results identifying independent predictors of candidemia following LDLT. Prolonged intubation (OR = 1.07; *p* = 0.019), bile leaks (OR = 10.9; *p* = 0.002), anidulafungin use (OR = 4.70; *p* = 0.032), fluconazole use (OR = 35.8; *p* = 0.005), and absence of CMV prophylaxis (OR = 11.7; *p* = 0.021) were independently associated with a higher likelihood of candidemia.

### 3.3. Comparison of Survivor and Non-Survivor Groups

Table 4 and Table 5 summarize the comparison of demographic, clinical, and pharmacological characteristics between survivors (n = 89) and non-survivors (n = 19) following LDLT. Among continuous variables, the duration of postoperative intubation was significantly longer in non-survivors [median 12 days (95% CI = 8–20)] compared with survivors [2 days (95% CI = 2–5)]; *p* < 0.001). No statistically significant differences were observed for other perioperative parameters, including age (*p* = 0.818), MELD score (*p* = 0.642), total hospital stay (*p* = 0.345), operation time (*p* = 0.474), cold ischemia time (*p* = 0.292) and warm ischemia time (*p* = 0.158).

For categorical variables, mortality was strongly correlated with the presence of candidemia (63.2% vs. 27%, OR = 4.6, *p* = 0.006), neutropenia (15.8% vs. 2.2%, OR = 8.2, *p* = 0.037), and chronic renal disease (21.1% vs. 0%, OR = 52, *p* = 0.001). Use of broad-spectrum antimicrobials, including meropenem (78.9% vs. 49.4%, OR = 3.8, *p* = 0.036), tigecycline (47.4% vs. 15.7%, OR = 4.8, *p* = 0.005), third-generation cephalosporins (21.1% vs. 1.1%, OR = 23.5, *p* = 0.003), and colistimethate sodium (42.1% vs. 7.9%, OR = 8.5, *p* < 0.001), was significantly associated with increased mortality. Similarly, antifungal therapy with anidulafungin (57.9% vs. 25.8%, OR = 3.9, *p* = 0.014) and fluconazole (21.1% vs. 5.6%, OR = 4.5, *p* = 0.049) was also significantly associated with higher mortality rates.

Furthermore, the lack of CMV prophylaxis (68.4% vs. 93.3%, OR = 6.3, *p* = 0.006) and positive CMV PCR results (26.3% vs. 5.6%, OR = 6.0, *p* = 0.014) were linked to significantly associated with higher mortality rates. Hemodialysis requirement was markedly higher among non-survivors (89.5% vs. 12.4%, OR = 60, *p* < 0.001), underscoring the impact of renal impairment and infectious complications on post-transplant mortality.

Among patients who did not survive within the candidemia group, *C. albicans* and *C. glabrata* were each identified in four cases, *C. parapsilosis* in two cases, and *C. kefir* and *C. tropicalis* in one case each. However, no specific *Candida* species demonstrated a statistically significant association with mortality within the candidemia group (*p* = 0.824).

## 4. Discussion

Opportunistic infections remain a major cause of morbidity and mortality following LT. The profound immunosuppression resulting from both the progression of end-stage liver disease and the administration of potent immunosuppressive agents to prevent graft rejection predisposes recipients to a wide range of post-transplant infections—including bacterial, viral, and fungal pathogens—as well as reactivation of latent infections such as CMV, Epstein–Barr Virus (EBV), Herpes Simplex Virus (HSV), Varicella-Zoster Virus (VZV), Mycobacterium tuberculosis and fungal infections [42,43,44]. This study aims to investigate clinical variables associated with the development of candidiasis, a major component of invasive fungal infections following LDLT, which holds both clinical relevance and statistical significance.

Opportunistic infections affect up to 80% of LT recipients. Bacterial infections are most common (70%) during the first postoperative month, followed by viral (20%) and fungal (8%) infections, which become more prevalent between one and six months post-transplant [42]. Among these, the overall incidence of invasive fungal infections among LT recipients remains substantial, with reported rates ranging from 4% to 42% [5,45,46]. *Candida* species are the leading main cause of invasive fungal infections in LT recipients, responsible for 68–80% of LT cases. The prevalence of candidiasis among invasive fungal infections also varies by transplant type—ranging from 49 to 60% in kidney, 38–68% in heart, 76% in pancreas, and up to 85% in small bowel transplants—while it is notably lower in lung transplants (23–47%) due to the predominance of *Aspergillus* species [5,13,45,47].

The distribution of *Candida* species is influenced by transplant type, antifungal prophylaxis, and geographic factors. *C. albicans* remains the most commonly isolated species in solid organ transplant recipients (30–74%), followed by *C. glabrata*, *C. parapsilosis*, *C. tropicalis*, and *C. krusei* [12,13,47,48]. In the present study, the distribution of *Candida* species was consistent with previous literature, with *C. albicans* identified as the predominant isolate, accounting for 41.7% of bloodstream infections among LDLT recipients.

The clinical impact of invasive fungal infections is considerable, with mortality rates reported between 25% and 80%, depending on the pathogen involved, with the highest rates observed in cases of invasive aspergillosis [5,12,13,45,46,48]. In the present study, approximately one-third of the patients with candidemia died during their hospital stay, and among those who died, *C. albicans* accounted for one-third of the isolates. When the overall outcomes are evaluated, the mortality rate was significantly higher in the candidemia group compared to the non-candidemia group, with 4.6-fold higher odds of mortality, reinforcing previous evidence regarding the severity of candidemia in LT recipients.

Identifying clinical variables associated with increased odds of developing invasive fungal infections after LT and comparing them with existing literature remains essential for guiding strategies to prevent or mitigate these opportunistic infections in future clinical practice. Senoner et al. [5] divided the risk factors for invasive fungal infections after LT into three main groups: (i) preoperative factors (MELD > 30, fungal colonization, preexisting renal failure, hospitalization, antimicrobial treatment), (ii) intraoperative factors (donor-derived infection, prolonged operation time, massive transfusion, urgent LT, bilioenteric anastomosis), and (iii) postoperative factors (renal replacement therapy, broad-spectrum antibiotics, early retransplantation, relaparotomy, CMV viremia, biliary leakage, prolonged intubation, TPN, immunosuppression, and central venous catheters). In the present study, the univariable analyses were consistent with many of the variables previously associated with increased odds of candidemia, as reported by Senoner et al. [5]. However, the present study demonstrated that both CMV prophylaxis and MMF use were associated with a reduced incidence of candidemia. Beyond these findings, the multivariable analysis confirmed that CMV prophylaxis was independently associated with a significantly lower risk of candidemia. Moreover, the effect sizes measured in the univariate analysis for both variables were within the moderate range, indicating meaningful clinical relevance alongside statistical significance. Notably, the association between MMF use and reduced odds of candidemia has not been consistently supported in previous LDLT studies and therefore warrants further investigation in future multicentric research.

Liu et al. [49] conducted a meta-analysis and found that various factors, including relaparotomy (OR = 2.2), dialysis after LT (OR = 2.0), bacterial infection (OR = 1.8), retransplantation (OR = 2.5), and fungal colonization (OR = 2.60), independently increased the risk of invasive fungal infections after LT. Phoompoung et al. [16] published a meta-analysis and showed that previous antibiotic use (OR = 9.3) and bacterial infection (OR = 4.3) were independent risk factors. However, when the authors specifically analyzed the last 10 years, they showed that previous fungal colonization (OR = 9.2), reoperation (OR = 5.5), and previous bacterial infections (OR = 3.8) were independent risk factors. The authors looked more closely at invasive aspergillosis and found that CMV (OR = 6.2), renal replacement therapy (OR = 9.2), and reoperation (OR = 8.0) were all separate risk factors. Jiang et al. [14] indicated that recipient age ≥ 55 years (OR = 2.7), MELD ≥ 22 (OR = 2.7), pretransplant elevated white blood cell (OR = 2.5), significant intraoperative blood loss (OR = 2.7), prolonged urethral catheter (OR = 3.2), and renal replacement therapy (OR = 5.8) were independently associated with the development of posttransplant invasive fungal infections. The research results also indicated that prophylactic antifungal therapy ≥ 3 days was associated with a lower risk of developing invasive fungal infections (OR = 0.16). When the results of all three studies are considered collectively, they appear to be consistent with the findings of the present study. However, the results reported by Jiang et al. [14] regarding age, MELD score, and WBC count did not show any correlation with either the current study or the three systematic reviews mentioned above. Therefore, we believe that further prospective multicentric studies are warranted to clarify this issue.

The present study found no specific association between candidemia and the primary immunosuppressive agents—tacrolimus, everolimus, and cyclosporine—or their blood concentrations, consistent with previous research [50]. However, the role of MMF remains controversial, as the limited available studies have yielded conflicting results. Anees et al. [51] reported that MMF, a widely used adjunct immunosuppressant, increased the prevalence of candidiasis compared to other agents, whereas Tirado-Sánchez et al. [52] demonstrated in a multivariable analysis that MMF exerted a protective effect against *Candida* infection. Paterson et al. [53] reported that MMF use does not appear to be associated with a significantly increased risk of infection occurring after LT. Supporting these conflicting observations, Papon et al. [54] suggested that certain immunosuppressants, particularly MMF, may exert natural antifungal activity capable of altering fungal ecology and favoring resistant or rare species. Such selective pressure could partly explain the heterogeneous results reported across studies. The natural antifungal activity of MMF may suppress commensal or sensitive fungal species while allowing the proliferation of resistant isolates, potentially influencing infection dynamics among transplant recipients. The present study observed a higher use of MMF in the non-candidemia group, suggesting a potential protective role for this agent. Tirado-Sánchez et al. [52] stated that in vitro cultures of *C. albicans* demonstrate sensitivity to MMF, an immunosuppressive agent utilized for transplant rejection avoidance and autoimmune disease treatment; these agents also inhibit inosine monophosphate dehydrogenase, the therapeutic target of MMF, findings consistent with the present study [52]. However, given the limited evidence in the literature, further well-designed prospective studies are warranted to confirm these observations.

Ohkubo et al. [55] determined that the incidence of invasive fungal infections after LT increased independently of reoperation, posttransplant dialysis, and bacterial infection; however, they reported that CMV infection (*p* = 0.470) did not increase the risk of fungal infection. The authors also showed that CMV infection was not associated with mortality (*p* = 0.08). Reed et al. [56] found that chronic renal failure was an independent risk factor (OR = 3.6; *p* = 0.014) for invasive fungal infections in high-risk LT recipients. This finding is consistent with the results of the present study’s univariate analysis, which also revealed a significant association between chronic renal failure and candidemia. Lum et al. [57] demonstrated no relationship between CMV viremia (*p* = 1.000) and invasive fungal infections, whereas George et al. [58] identified CMV infection as an independent risk factor for invasive fungal infections in LT recipients (OR = 5.8; *p* = 0.003). In the meta-analysis conducted by Phoompoung et al. [16] covering the years 1990–2019, CMV infection was found to increase the odds of invasive fungal infections by 5.0-fold. However, in the subgroup analysis including data from the most recent nine years, this association was no longer observed, indicating that the relationship between CMV infection and invasive fungal infections may have diminished over time. Senoner et al. [5] similarly concluded that CMV infection is an independent risk factor for the development of invasive fungal infections, with odds ratios ranging from 4.8 to 9.4. According to Liu et al. [59], CMV represents a significant risk factor for infections caused by *Candida*, *Aspergillus*, and *Cryptococcus* species. Therefore, administering antiviral prophylaxis to LT recipients at high risk for CMV disease may significantly reduce the risk of candidemia, even in the absence of antifungal prophylaxis. Consistent with these findings and previous reports, CMV-PCR positivity was found to be significantly associated with candidemia in the present study [11,59,60]. The present study further showed that the candidemia group had 5.6-fold higher odds of CMV PCR positivity, whereas the non-candidemia group had 13.5-fold higher odds of receiving CMV prophylaxis. Hence, the early initiation of CMV prophylaxis may have a protective effect against the development of posttransplant candidemia.

Antifungal prophylaxis after LT carries potential risks such as drug toxicity, resistance development, and breakthrough infections. Consequently, fungal infections remain an active area of investigation and are expected to continue attracting research attention in the future [12]. Kim et al. [12] found in the univariate analysis that CMV (OR = 4.1; *p* = 0.001) and antifungal agents (OR = 13.6; *p* = 0.0006) were associated with invasive fungal infections, while in the multivariable analysis, the use of antifungal agents (OR = 28.5; *p* = 0.002) was identified as an independent risk factor—findings consistent with those of the present study. Raghuram et al. [61] reported that fluconazole prophylaxis at doses below 200 mg was associated with an increased risk of developing resistant strains causing invasive fungal infections (OR = 2.8; *p* = 0.03), highlighting the potential harm of low-dose antifungal prophylaxis. Likewise, Lum et al. [57] identified fluconazole prophylaxis as a risk factor for invasive fungal infections (OR = 2.9; *p* = 0.04), in line with the findings of the present study. In contrast, a meta-analysis published by Phoompoung et al. [16] involving LT recipients reported that antifungal prophylaxis reduced the risk of invasive fungal infections by 3.1-fold. The apparent discrepancy between these results and those of the present study, which identified antifungal use as a risk factor for candidemia, may be attributable to differences in study design, patient selection, antifungal dosing, and prophylaxis strategies.

LT recipients receiving antifungal prophylaxis frequently develop non-albicans candidemia and related side effects [62]. Such prophylaxis may promote resistance and the emergence of non-albicans species [63,64]. A multicenter open-label trial demonstrated that caspofungin increased the risk of invasive fungal infections in LT recipients, and several studies have described antifungal prophylaxis as controversial or potentially harmful [59]. The present study supports these findings, identifying antifungal therapy as an independent risk factor for candidemia. Conversely, some evidence suggests that postoperative fluconazole prophylaxis may reduce *Candida* colonization [65], although many studies have reported no significant difference between placebo and antifungal prophylaxis in preventing fungal infections after organ transplantation. Moreover, a large multicenter prospective study showed no mortality benefit from empirical antifungal therapy in transplant recipients [66].

The studies conducted in transplantation units identified broad-spectrum antibiotics, central venous catheters, TPN, and *Candida* colonization as risk factors for invasive candidiasis, which is compatible with the association between central venous catheters, broad-spectrum antibiotics, and candidemia in the present study [50,59,67,68]. Despite a previous study’s finding that ceftazidime plus amoxicillin was safer than ciprofloxacin plus amoxicillin in preventing fungal infections, the present study found that third-generation cephalosporins, but not quinolones, were associated with candidemia [69]. We can state that there is no consistency in the safety of ceftazidime or ciprofloxacin. Shi et al. [70] found postoperative bacterial infection to be an independent risk factor for the emergence of invasive *Candida* infections after LT. Another study identified the number of antibiotics, duration of antibiotic therapy, and bacteraemia as risk factors for invasive fungal infections in LT recipients [58]. From a similar perspective, the present study found a relationship between candidemia and various broad-spectrum antibacterial agents. In univariate analysis, linezolid use was associated with an approximately 4.5 times higher odds of candidemia (OR = 4.5); however, this association did not remain statistically significant in the multivariable analysis. Therefore, linezolid was not identified as an independent risk factor in the adjusted model. The research conducted by Falagas et al. [71] reported that linezolid may induce fungal infections as an adverse effect, a finding consistent with the results of the present study’s univariate analysis. Taken together, these studies suggest that further research is warranted to better understand the potential association between linezolid use and candidemia.

In the present study, prolonged intubation and bile leakage were identified as independent factors associated with an increased risk of candidemia following lLDLT. These findings emphasize the critical role of respiratory and biliary management in reducing posttransplant invasive fungal infections and, consequently, infection-related morbidity and mortality. Phoompoung et al. [16] conducted a comprehensive meta-analysis and reported that, in earlier cohorts, biliary complications increased the risk of invasive fungal infections by 3.6-fold. However, in their updated analysis including more recent data, no such association was observed, suggesting that advances in surgical techniques, early diagnosis, and targeted antifungal strategies may have mitigated this risk over time. Michalopoulos et al. [72] reported that when intubation exceeded 10 days, the odds of developing candidemia increased 127-fold, and mortality in the candidemia group was 28 times higher. These findings closely parallel the results of the present study.

Disseminated invasive fungal disease is relatively common among the LT recipients and is associated with a high mortality rate of around 65% [5,16]. In the present study, a secondary objective was to compare survivors and non-survivors following LDLT to identify mortality-associated factors. The analysis revealed that prolonged intubation, neutropenia, candidemia, chronic renal disease, hemodialysis, TPN use, lack of CMV prophylaxis, CMV PCR positivity, and the use of certain broad-spectrum antibiotics and antifungals were significantly associated with mortality. Among the 108 patients included in this study, 19 (17.6%) died, and candidemia was present in 63.2% of these non-survivors, findings that are consistent with previous studies demonstrating that infection-related complications remain the leading cause of early post-transplant mortality.

### 4.1. Limitations

The present study is subject to several important limitations that should be acknowledged when interpreting the findings. First, the retrospective case–control design inherently introduces a potential risk of selection and information bias. Although the control group was established using demographic and clinical matching criteria, residual confounding cannot be fully excluded. Second, the study’s single-center nature and relatively limited sample size may restrict the generalizability of the results to other transplant populations or institutional settings. Moreover, as LT is performed only in a few specialized centers in our country and there is no national registry for candidemia, the ability to perform a large-scale multicenter analysis was constrained. Finally, the retrospective nature of antimicrobial exposure assessment may have introduced minor inconsistencies in drug duration or timing data. Despite these limitations, the study provides valuable real-world evidence from one of the highest-volume LDLT centers, contributing to a better understanding of the epidemiological and clinical determinants of post-transplant candidemia.

### 4.2. Future Directions

The results of this study highlight several important areas for future research. Although the multivariable analysis did not confirm all associations observed in univariate analyses, two notable findings—an apparent link between HCC and candidemia, and a potential protective effect of MMF—warrant further exploration. The underlying mechanisms of these associations remain unclear and should be investigated in well-designed prospective multicentric studies with larger cohorts. Future research should also focus on the impact of CMV prophylaxis, which demonstrated a protective effect against candidemia in the present study, to validate whether antiviral prophylaxis independently mitigates the risk of fungal infection. Moreover, antifungal stewardship programs should be systematically evaluated to establish safe and effective prophylaxis protocols that minimize resistance development. Incorporating molecular diagnostics and surveillance-based risk stratification models may improve early detection and targeted prevention of invasive fungal infections in high-risk LDLT recipients. Collectively, these efforts could lead to the development of standardized, evidence-based guidelines for the prevention and management of candidemia in liver transplant populations.

## 5. Conclusions

The present study identified several clinical and pharmacological factors associated with the development of candidemia following LDLT. Univariate analysis demonstrated that prolonged intubation, bile leaks, relaparotomy, chronic renal disease, HCC, exposure to specific broad-spectrum antibiotics and antifungal agents, TPN, and CMV PCR positivity were all significantly associated with increased odds of candidemia. Conversely, CMV prophylaxis and the use of MMF were associated with a reduced risk. Multivariable logistic regression analysis confirmed that prolonged intubation, bile leaks, anidulafungin, and fluconazole were independent predictors of candidemia, whereas CMV prophylaxis exerted a protective effect. These findings underscore the importance of prudent antimicrobial stewardship, early detection and management of biliary complications, and the judicious use of antifungal prophylaxis. Furthermore, the results suggest that excessive or unnecessary antifungal exposure may be detrimental, while timely CMV prophylaxis could play a protective role. Overall, the implementation of targeted infection control strategies, optimization of respiratory and biliary management, and individualized prophylactic regimens may substantially reduce infection-related morbidity and mortality among LDLT recipients.

## Figures and Tables

**Table 1:** Comparison of continuous demographic and perioperative parameters between LDLT recipients with and without candidemia.

Variables [Median (95% CI)]	Candidemia (n = 36)	Non-Candidemia (n = 72)	Cohen’s d	*p*
Age (years)	54 (50–59)	55 (52–59)	0.00	0.860
MELD score	18 (14–24)	16 (14–18)	0.14	0.463
Hospital stay (Pre LT; days)	2 (2–6)	2 (2–5)	0.10	0.840
Hospital stay (Post LT; days)	36 (32–42)	40 (37–45)	0.30	0.091
Operation time (min)	480 (480–540)	500 (480–540)	0.14	0.650
CIT (min)	44 (43–46)	45 (43–48)	0.13	0.737
WIT (min)	52 (48–55)	54 (48–59)	0.00	0.863
Intraop crystalloids (ml)	6200 (5700–7300)	6000 (5500–6500)	0.08	0.622
Prolonged intubation	8 (4–16)	2 (2–5)	0.68	<0.001
Tacrolimus level	6 (4–8)	5 (5–7)	1.16	0.691

CIT: Cold ischemia time; WIT: Warm ischemia time.

**Table 2:** Comparison of categorical postoperative complications, antimicrobial exposure, and immunosuppressive variables between LDLT recipients with and without candidemia.

Variables	Candidemia (n = 36)	Non-Candidemia (n = 72)	Cramer’s V	OR	*p*
Gender (Male; %)	23 (63.9)	47 (65.3)	0.01		1.000
Ascites (Pre LT) (%)	21 (58.3)	32 (44.4)	0.13		0.247
IOBT (%)	11 (30.6)	27 (37.5)	0.07		0.618
HJ	5 (13.9)	7 (9.7)	0.06		0.529
Bile leaks	26 (72.2)	23 (31.9)	0.38	5.5	<0.001
Relaparotomy	23 (63.9)	17 (23.6)	0.39	5.7	<0.001
Neutropenia (%)	3 (8.3)	2 (2.8)	0.13		0.331
**Comorbidity (n; %)**					
DM	14 (38.9)	19 (26.4)	0.13		0.268
HT	10 (27.8)	21 (29.2)	0.01		1.000
CAD	6 (16.7)	9 (12.5)	0.06		0.768
CRD *	4 (11.1)	0 (0.0)	0.28	11.1	0.011
HCC *	4 (11.1)	0 (0.0)	0.28	11.1	0.011
COPD	2 (5.6)	4 (5.6)	0.00		1.000
**Antibiotics (n; %)**					
Ampicillin-sulbactam	13 (36.1)	22 (30.6)	0.06		0.716
Meropenem	25 (69.4)	34 (47.2)	0.21	2.5	0.048
Ertapenem	3 (8.3)	17 (23.6)	0.19		0.096
Imipenem	4 (11.1)	7 (9.7)	0.02		1.000
Linezolid	13 (36.1)	8 (11.1)	0.30	4.5	0.005
Piperacillin-tazobactam	12 (33.3)	22 (30.6)	0.03		0.942
Tigecycline	12 (33.3)	11 (15.3)	0.21	2.8	0.045
Glycopeptides	10 (27.8)	18 (25.0)	0.03		0.938
Colistimethate sodium	8 (22.2)	7 (9.7)	0.17		0.086
3rd generation cephalospororine	5 (13.9)	0 (0.0)	0.31	13.7	0.003
Aminoglycosides	5 (13.9)	5 (6.9)	0.11		0.203
Quinolones	2 (5.6)	2 (2.8)	0.07		0.599
Metronidazole	1 (2.8)	2 (2.8)	0.00		1.000
**Antifungals (n; %)**					
Anidulafungin	20 (55.6)	14 (19.4)	0.37	5.2	<0.001
Fluconazole	7 (19.4)	2 (2.8)	0.28	8.5	0.006
Amphotericin B	2 (5.6)	2 (2.8)			0.599
**Immunosuppressive (n; %)**					
Tacrolimus	34 (94.4)	69 (95.8)	0.03		1.000
MMF *	32 (88.9)	72 (100)	0.28	11.1	0.011
Everolimus	5 (13.9)	12 (16.7)	0.04		0.926
Cyclosporine	1 (2.8)	3 (4.2)	0.04		1.000
**Others (n; %)**	13 (36.1)	15 (20.8)			0.140
Hemodialysis (n; %)	13 (36.1)	15 (20.8)			0.140
TPN (n; %)	16 (44.4)	18 (25.0)	0.20	2.4	0.049
CMV prophylaxis (n; %)	26 (72.2)	70 (97.2)	0.38	13.5	<0.001
CMV PCR positive (n; %)	7 (19.4)	3 (4.2)	0.25	5.6	0.015
Mortality (n; %)	12 (33.3)	7 (9.7)	0.29	4.6	0.006

* OR and 95% CI were calculated using the Haldane-Anscombe correction, which was applied to address zero-cell counts in contingency tables. CAD: Coronary artery disease; CMV: Cytomegalovirus; CRD: Chronic renal disease; DM: Diabetes mellitus; HCC: Hepatocellular carcinoma; HJ: Hepaticojejunostomy; HT: Hypertension; IOBT: Intraoperative blood transfusion; LT: Liver transplantation; MMF: Mycophenolate mofetil; PCR: Polymerase chain reaction; TPN: Total parenteral nutrition.

**Table 3:** Multivariable logistic regression analysis identifying independent predictors of candidemia after LDLT.

Predictive Factors	B	SE	Wald	*p*	OR	95% CI
Prolonged intubation	+0.066	0.028	5.543	0.019	1.07	1.01–1.13
Bile Leaks	+2.388	0.783	9.300	0.002	10.9	2.35–50.5
Anidulafungin	+1.549	0.721	4.612	0.032	4.70	1.5–19.3
Fluconazole	+3.580	1.279	7.831	0.005	35.8	2.9–440
CMV prophylaxis	−2.463	1.070	5.301	0.021	11.7	1.44–100

Hosmer and Lemeshow significance level 0.934 (chi square = 3.01).

**Table 4:** Comparison of survivor and non-survivor groups in terms of some demographic and clinical continuous variables.

Variables [Median (95% CI)]	Survivor (n = 89)	Non-Survivor (n = 19)	Cohen’s d	*p*
Age (years)	55 (52–58)	52 (50–59)	0.00	0.818
MELD score	16 (14–18)	16 (14–21)	0.00	0.642
Hospital stay (Pre LT; days)	2 (2–4)	2 (2–7)	0.25	0.345
Hospital stay (Post LT; days)	38 (36–42)	42 (27–61)	0.22	0.692
Operation time (min)	500 (490–540)	480 (480–565)	0.17	0.474
CIT (min)	45 (43–48)	44 (40–46)	0.26	0.292
WIT (min)	52 (50–56)	58 (45–74)	0.44	0.158
Intraop crystalloids (ml)	6100 (5700–6500)	5200 (5000–7000)	0.40	0.137
Prolonged intubation	2 (2–5)	12 (8–20)	0.70	<0.001
Tacrolimus level	5 (5–7)	7 (3–10)	0.56	0.120

CIT: Cold ischemia time; WIT: Warm ischemia time; MELD: Model for end-stage liver disease; LT: liver transplantation.

**Table 5:** Comparison of survivor and non-survivor groups in terms of clinical categorical variables.

Variables	Survivor (n = 89)	Non-Survivor (n = 19)	Cramer’s V	OR	*p*
Gender (Male; %)	59 (66.3)	11 (57.9)	0.07		0.598
Ascites (Pre LT) (%)	44 (49.4)	9 (47.4)	0.02		1.000
IOBT (%)	33 (37.1)	5 (26.3)	0.09		0.531
Hepaticojejunostomy (%)	10 (11.2)	2 (10.5)	0.01		1.000
Bile leaks (%)	40 (44.9)	9 (47.4)	0.02		1.000
Relaparotomy (%)	30 (33.7)	10 (52.6)	0.15		0.197
Neutropenia (%)	2 (2.2)	3 (15.8)	0.25	8.2 (1.3–52.8)	0.037
Candidemia (%)	24 (27)	12 (63.2)	0.29	4.6 (1.6–13.2)	0.006
**Comorbidity (n; %)**					
DM	25 (28.1)	8 (42.1)	0.12		0.353
HT	24 (27)	7 (36.8)	0.08		0.559
CAD	11 (12.4)	4 (21.1)	0.10		0.297
CRD	0 (0)	4 (21.1)	0.42	52 (3–1014)	0.001
HCC	2 (2.2)	2 (10.5)	0.17		0.142
**Antibiotics (n; %)**					
Ampicillin-sulbactam	30 (33.7)	5 (26.3)	0.06		0.723
Meropenem	44 (49.4)	15 (78.9)	0.23	3.8 (1.2–12.5)	0.036
Ertapenem	20 (22.5)	0 (0)	0.22		0.021
Imipenem	10 (11.2)	1 (5.3)	0.08		0.685
Linezolid	15 (16.9)	6 (31.6)	0.14		0.198
Piperacillin-tazobactam	28 (31.5)	6 (31.6)	0.01		1.000
Tigecycline	14 (15.7)	9 (47.4)	0.29	4.8 (1.7–14)	0.005
Glycopeptides	23 (25.8)	5 (26.3)	0.01		1.000
Colistimethate sodium	7 (7.9)	8 (42.1)	0.38	8.5 (2.6–28)	<0.001
3rd generation cephalos	1 (1.1)	4 (21.1)	0.36	23.5 (2.5–225)	0.003
Aminoglycosides	7 (7.9)	3 (15.8)	0.10		0.376
Quinolones	3 (3.4)	1 (5.3)	0.04		0.544
Metronidazole	3 (3.4)	0 (0)	0.08		1.000
**Antifungals (n; %)**					
Anidulafungin	23 (25.8)	11 (57.9)	0.26	3.9 (1.4–11)	0.014
Fluconazol	5 (5.6)	4 (21.1)	0.21	4.5 (1.1–18.6)	0.049
Amphotericin B	3 (3.4)	1 (5.3)	0.04		0.544
**Immunosuppressive (n; %)**					
Tacrolimus	85 (95.5)	18 (94.7)	0.01		1.000
MMF	86 (96.6)	18 (94.7)	0.04		0.544
Everolimus	13 (14.6)	4 (21.1)	0.07		0.495
Cyclosporine	4 (4.5)	0 (0)	0.09		1.000
**Others (n; %)**					
Hemodialysis (n; %)	11 (12.4)	17 (89.5)	0.67	60 (12–297)	<0.001
TPN (n; %)	24 (27)	10 (52.6)	0.21	3 (1.1–8.3)	0.056
CMV prophylaxis (n; %)	83 (93.3)	13 (68.4)	0.30	6.3 (1.8–22.7)	0.006
CMV PCR positive (n; %)	5 (5.6)	5 (26.3)	0.27	6 (1.5–23.4)	0.014

CAD: Coronary artery disease; CMV: Cytomegalovirus; CRD: Chronic renal disease; DM: Diabetes mellitus; HCC: Hepatocellular carcinoma; HJ: Hepaticojejunostomy; HT: Hypertension; IOBT: Intraoperative blood transfusion; LT: Liver transplantation; MMF: Mycophenolate mofetil; PCR: Polymerase chain reaction; TPN: Total parenteral nutrition.

## Data Availability

The data presented in this study are available upon request from the corresponding author.
